# Medical Diagnosis Reimagined as a Process of Bayesian Reasoning and Elimination

**DOI:** 10.7759/cureus.45097

**Published:** 2023-09-12

**Authors:** Amogh Ananda Rao, Milind Awale, Sissmol Davis

**Affiliations:** 1 Quantitative Biology and Bioinformatics, Carnegie Mellon University, Pittsburgh, USA; 2 Internal Medicine, Wheeling Hospital, Wheeling, USA; 3 Internal Medicine, JJM Medical College, Davangere, IND

**Keywords:** holistic care for patients, machine leaning, kartagener's syndrome, bayes theorem, artificial intelligence in medicine, art of diagnosis

## Abstract

This article delves into the interface between the art of medical diagnosis and the mathematical foundations of probability, the Bayes theorem. In a healthcare ecosystem witnessing an artificial intelligence (AI)-driven transformation, understanding the convergence becomes crucial for physicians. Contrary to viewing AI as a mysterious “black box,” we demonstrate how every diagnostic decision by a medical practitioner is, in essence, Bayesian reasoning in action. The Bayes theorem is a mathematical translation of systematically updating our belief: it quantifies how an additional piece of information updates our prior belief in something. Using a clinical scenario of Kartagener syndrome, we showcase the parallels between a physician’s evolving diagnostic thought process and the mathematical updating of prior beliefs with new evidence. By reimagining medical diagnosis through the lens of Bayes, this paper aims to demystify AI, accentuating its potential role as an enhancer of clinical acumen rather than a replacement. The ultimate vision presented is one of harmony, where AI serves as a symbiotic partner to physicians, with the shared goal of holistic patient care.

## Introduction

We are in a transformative phase in the history of medical science. The recent advances in artificial intelligence (AI) promise a revolution in the way medicine of the future will be practiced. Physicians world over are poised somewhere between anticipation and apprehension of what is to come. The seeming complexity, “black-box” nature of many AI systems, and ethical and medico-legal considerations loom large over the potential integration of AI in healthcare [1]. For medical wisdom and modern technology to converge in harmony, it is important for physicians to develop a basic understanding and a sense of trust in the technological addendum. Of course, AI cannot replace the intuition, empathy, and nuanced understanding of seasoned medical professionals, but the goal is a symbiosis where technology amplifies the physician’s capabilities, and traditional medical wisdom guides the ethical and appropriate use of technology.

This article is not just an insight but an invitation to view the art of medical diagnosis through the lens of mathematics. Making sense of large amounts of data requires the science of probability. As Sir William Osler put it, “Medicine is a science of uncertainty and an art of probability.” The Bayes theorem is a foundational pillar of probability and provides a mechanism for us to mathematically update our beliefs in light of new evidence [2]. What makes the Bayes theorem powerful is the realization that our beliefs are not static; they evolve as we are presented with more and more information.

## Technical report

The Bayes theorem features repeatedly in machine learning (ML), especially where we deal with models that need constant refinement. Implicitly or explicitly, it plays a crucial role in natural language processing, neural networks, and many other ML models. To reimagine the art of medical diagnosis through the Bayes theorem provides insight and intuition for physicians to understand ML principles and applications.

Every new patient walking into the consultation room is a puzzle waiting to be solved. The symptoms they describe and their medical history provide information for our initial belief. The physician, with years of training and experience, drafts a preliminary list of probable conditions: a differential diagnosis. However, to zero in on a single diagnosis, we progressively eliminate items on the differential based on the new evidence we discover based on the physical exam and investigations we order. Each additional test, every nuanced symptom, and even external factors like epidemiological data play their part in this evolution. As we mentally adjust the probabilities, certain diagnoses gain prominence, while others fade into the background. This clinical intuition is just Bayesian reasoning in action.

**Figure 1:**
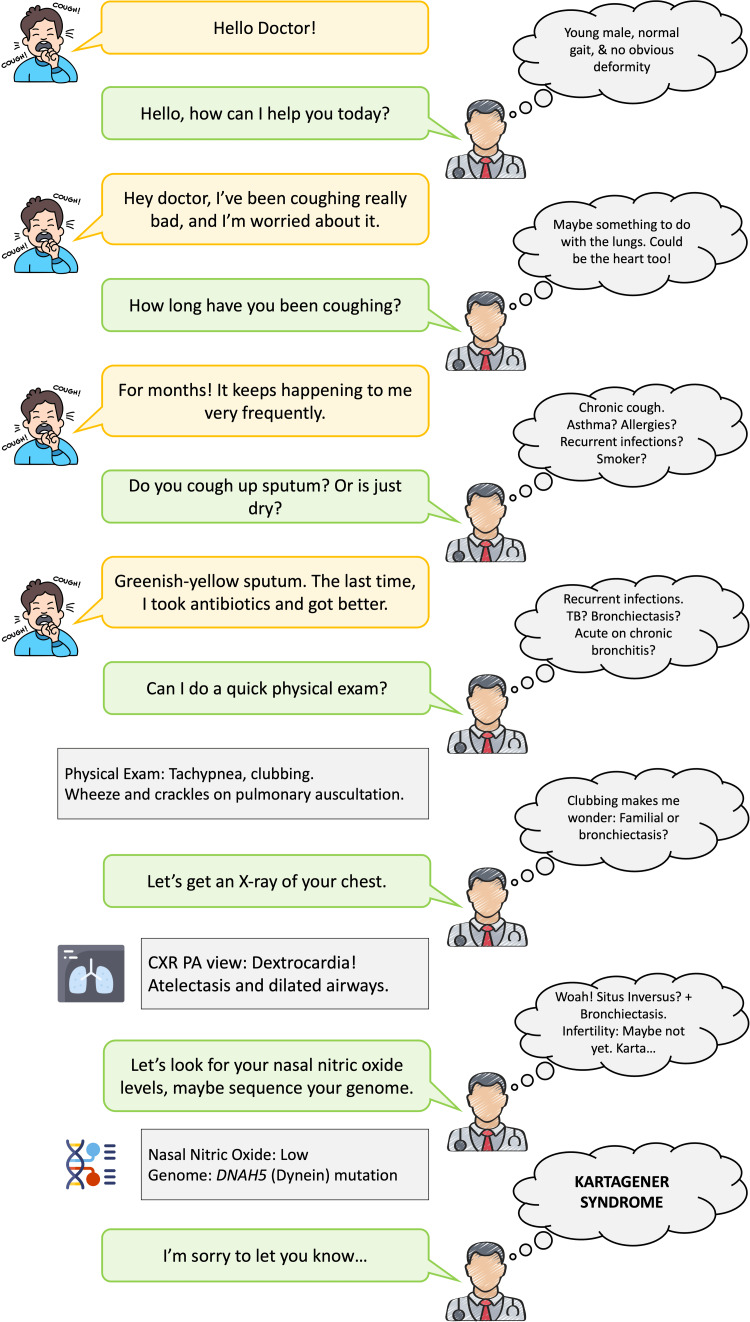
Bayesian reasoning in medical diagnosis

Imagine you are in an outpatient clinic, and the next patient presents to you with a cough for many months. The scenario in Figure 1 provides a perfect example of how the Bayes theorem works.

The art of diagnosis is a classic and intuitive way to understand the Bayes theorem. (Let’s also excuse our doctor for not strictly adhering to the best practices.) Note: Kartagener syndrome (KS) is a rare, autosomal recessive disease affecting ciliary function. The patients generally have a triad of situs inversus, chronic recurrent sinusitis, and bronchiectasis.

## Discussion

In the language of mathematics, the initial belief we start with is known as the “prior probability,” and as the new evidence updates our belief, we call it the “posterior probability.” In this example, our initial belief that the patient has KS is the prior. When we receive new information, or “data,” we update our belief, which becomes the posterior. This update can strengthen, weaken, or leave our belief unchanged.

**Figure 2:**
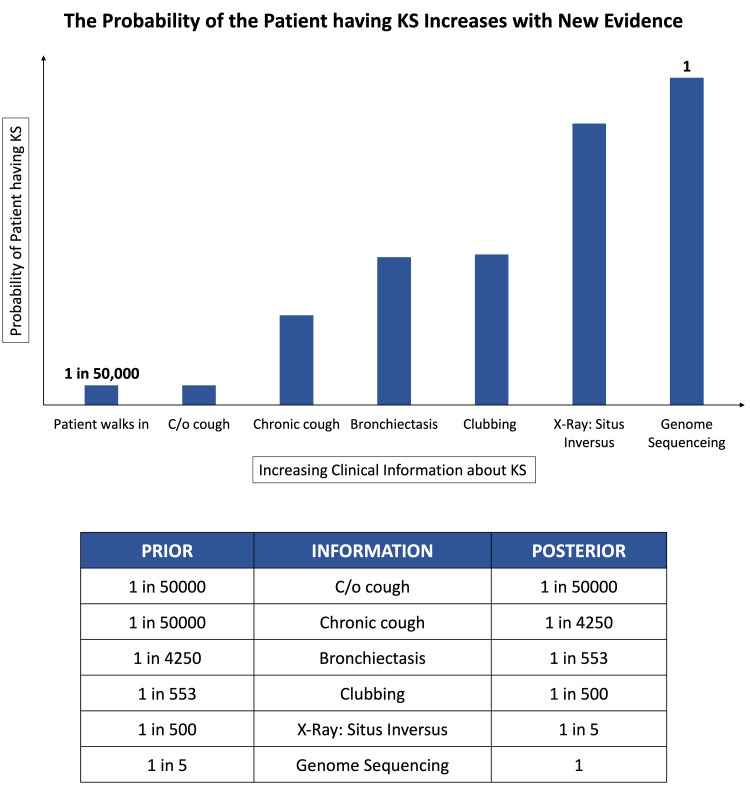
Kartagener syndrome diagnosis: a Bayesian perspective

Initially, the likelihood of the patient having KS is equivalent to its prevalence in the general population, which is one in 50,000 [3]. The patient’s cough, being a common symptom, doesn’t significantly alter this initial probability. However, when the cough is identified as chronic, the probability of the patient having KS increases to one in 4,250 [4], reinforcing our initial suspicion. Further evidence of bronchiectasis strengthens our belief that the patient could have KS [5,6]. When we learn that the patient also has situs inversus, the likelihood of a KS diagnosis dramatically increases to one in five [7]. Finally, genome sequencing confirms that the patient has a mutation in the dynein protein, a key component of cilia [8]. At this stage, we can definitively diagnose the patient with KS. In summary, as we gather more evidence, our level of certainty regarding a KS diagnosis gradually increases, until it becomes a confirmed fact. The progressive increase in the likelihood of the patient having KS, based on accumulating evidence, is graphically presented in Figure 2.

The graph illustrates how our confidence in diagnosing the patient with KS evolves as we consider each new piece of information. The table provides a detailed summary, showing step-by-step changes in both our initial (prior) and updated (posterior) probabilities that the patient has KS. In essence, the table tracks how each new piece of information affects our evolving belief about the likelihood of a KS diagnosis. (Note: The graph has been logarithmically scaled by a base of 106 for better visualization).

## Conclusions

Modern medicine is woven with threads of clinical acumen, empathy, technological advances, and mathematical reasoning. As the prominence of AI grows, understanding its underpinnings becomes paramount. Medical diagnosis showcases Bayesian reasoning in all its glory. Recognizing this not only demystifies AI but also emphasizes the harmonious coexistence of intuitive human judgment with computational prowess.

The convergence of medicine and mathematics offers an exciting future where AI, far from being a competitor, is an ally, amplifying the physician’s capabilities and ensuring that the patient remains at the heart of the healing process.

## Appendices

The Bayes theorem

The Bayes theorem is mathematically represented as:

$P(A|B) = \frac{P(A) \times P(B|A)}{P(B)}$

where *P(A)* is the prior(initial) probability of belief *A* being true, before considering the new evidence *B*; *P(B)* is the probability that evidence *B* is true; *P(A|B)* is the posterior(updated) probability of *A* being true, given that evidence *B* is true; *P(B|A)* is the probability of observing evidence *B*, given that hypothesis *A* is true.

*A *is our belief that the patient has Kartagener syndrome(KS), and *B *is every new piece of clinical information we use for the diagnosis. To add some context to this formula, we can rewrite it as, 

$P(KS|Info) = \frac{P(KS) \times P(Info|KS)}{P(Info)}$

**Table 1:** Using Bayes theorem to diagnose Kartagener syndrome

Information	P(Info)	P(KS)	P(Info|KS)	P(KS|Info)	P(KS|Info) in words	Reference
Patient walks in	1	0.00002	1	0.00002	1 in 50000	3
C/o cough	1	0.00002	1	0.00002	1 in 50000	-
Chronic cough	0.085	0.00002	1	0.00024	1 in 4250	4
Bronchiectasis	0.01106	0.00002	1	0.00181	1 in 553	5
Clubbing	0.01	0.00002	1	0.00200	1 in 500	6
X-ray: situs inversus	0.0001	0.00002	1	0.20000	1 in 5	7
Genome sequencing	0.00002	0.00002	1	1.00000	1	8

P(KS) means the probability of a patient having KS, which is the same as the prevalence of KS in the population. P(Info) is the probability of the piece of information being true; for instance, the prevalence of situs inversus is 0.0001. P(Info|KS) is the probability of the piece of information being true when it is given that the patient has KS; for instance, every patient with KS has situs inversus, and thus the value is 1. P(KS|Info) reflects the art of diagnosis: it is the probability that the patient does have KS when the piece of information is given to us. Thus, we can estimate that the probability of the patient with a finding of situs inversus having KS is 0.2 (one in five).
